# The Flagellar Gene Regulates Biofilm Formation and Mussel Larval Settlement and Metamorphosis

**DOI:** 10.3390/ijms21030710

**Published:** 2020-01-21

**Authors:** Xiao Liang, Xiu-Kun Zhang, Li-Hua Peng, You-Ting Zhu, Asami Yoshida, Kiyoshi Osatomi, Jin-Long Yang

**Affiliations:** 1 International Research Center for Marine Biosciences, Ministry of Science and Technology, Shanghai Ocean University, Shanghai 201306, China; x-liang@shou.edu.cn (X.L.); m170100004@st.shou.edu.cn (X.-K.Z.); d170103035@st.shou.edu.cn (L.-H.P.); ytzhu@shou.edu.cn (Y.-T.Z.); 2 Key Laboratory of Exploration and Utilization of Aquatic Genetic Resources, Ministry of Education, Shanghai Ocean University, Shanghai 201306, China; 3 National Demonstration Center for Experimental Fisheries Science Education, Shanghai Ocean University, Shanghai 201306, China; 4 Graduate School of Fisheries and Environmental Sciences, Nagasaki University, Nagasaki 8528521, Japan; y-asami@nagasaki-u.ac.jp (A.Y.); osatomi@nagasaki-u.ac.jp (K.O.)

**Keywords:** mussel, *Mytilus coruscus*, larval settlement and metamorphosis, biofilm, flagellar gene

## Abstract

Biofilms are critical components of most marine systems and provide biochemical cues that can significantly impact overall community composition. Although progress has been made in the bacteria–animal interaction, the molecular basis of modulation of settlement and metamorphosis in most marine animals by bacteria is poorly understood. Here, *Pseudoalteromonas marina* showing inducing activity on mussel settlement and metamorphosis was chosen as a model to clarify the mechanism that regulates the bacteria–mussel interaction. We constructed a flagellin synthetic protein gene *fliP* deletion mutant of *P. marina* and checked whether deficiency of *fliP* gene will impact inducing activity, motility, and extracellular polymeric substances of biofilms. Furthermore, we examined the effect of flagellar proteins extracted from bacteria on larval settlement and metamorphosis. The deletion of the *fliP* gene caused the loss of the flagella structure and motility of the Δ*fliP* strain. Deficiency of the *fliP* gene promoted the biofilm formation and changed biofilm matrix by reducing β-polysaccharides and increasing extracellular proteins and finally reduced biofilm-inducing activities. Flagellar protein extract promoted mussel metamorphosis, and Δ*fliP* biofilms combined with additional flagellar proteins induced similar settlement and metamorphosis rate compared to that of the wild-type strain. These findings provide novel insight on the molecular interactions between bacteria and mussels.

## 1. Introduction

In the marine environment, biofilms are critical components of most marine systems and provide biochemical cues that can significantly impact overall community composition [1,2]. Larval settlement and metamorphosis of many invertebrate species are regulated by biofilms that are universally present on biotic and abiotic surfaces [3,4,5]. Biofilm development has been known to be mediated by cellular and environmental factors, and the production of settlement-inducing cues of biofilms also varies due to the space-time change in biofilm characteristics including cell density and structure [6,7,8].

Marine bacteria dominate in the natural biofilms developed in ocean conditions [9,10,11]. *Pseudoalteromonas* is widely distributed in marine environments [12], and biofilms formed by *Pseudoalteromonas* mediate settlement and metamorphosis of many typical macrofouling organisms, including mussel, barnacle, and tubeworm [13,14,15,16]. Although some progress has been made in the specific animal *Hydroides elegans* [15,16,17,18], the molecular basis of modulation of settlement and metamorphosis in most marine animals by bacteria is poorly understood. 

*Mytilus coruscus*, is a major economic shellfish and typical macrofouling organism in the East China Sea [14,19,20]. Our recent studies demonstrated that three genes in *Pseudoalteromonas,* namely *AT00-08765*, *AT00-17125,* and *hmgA* are involved in motility or production of cellulose, capsular polysaccharide, and extracellular pyomelanin and cause the phenotype variance, and biofilms formed by three mutants simultaneously inhibite larval settlement and metamorphosis in *M. coruscus* [21,22]. Here, we used a *Pseudoalteromonas marina* [23] as a model to clarify the mechanism that regulates the biofilm–mussel interaction. We constructed a flagellin synthetic protein gene *fliP* deletion mutant of *P. marina* and tested whether deficiency of *fliP* gene impacts inducing activity, motility, and extracellular polymeric substances (EPS) of biofilms. In addition, we also extracted the flagellar protein and examined the effect of flagellar proteins on mussel settlement and metamorphosis.

## 2. Results

### 2.1. Deletion of fliP Gene Reduced Mussel Larval Settlement and Metamorphosis

**Figure 1:**
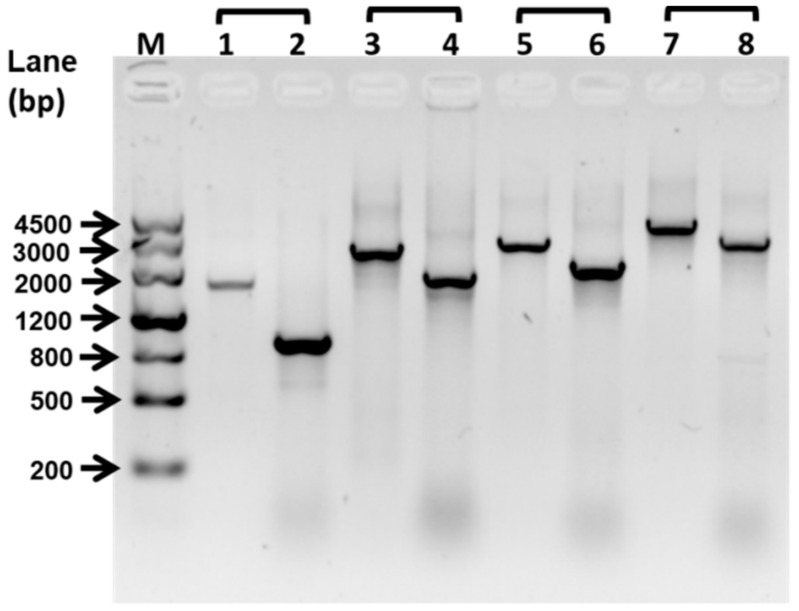
Deletion of flagellin synthetic protein gene *fliP*. The deletion of the *fliP* gene was confirmed by PCR. The PCR product sizes of the wild-type strain were 1637 bp (line 1, *fliP*–SF/*fliP*–SR), 2558 bp (line 3, *fliP*–SF/*fliP*–LR), 2805 bp (line 5, *fliP*–LF/*fliP*–SR), and 3726 bp (line 7, *fliP*–LF/*fliP*–LR). The PCR product sizes of Δ*fliP* strain were 896 bp (line 2, *fliP*–SF/*fliP*–SR), 1817 bp (line 4, *fliP*–SF/*fliP*–LR), 2064 bp (line 6, *fliP*–LF/*fliP*–SR), and 2985 bp (line 7, *fliP*–LF/*fliP*–LR). M, marker.

**Figure 2:**
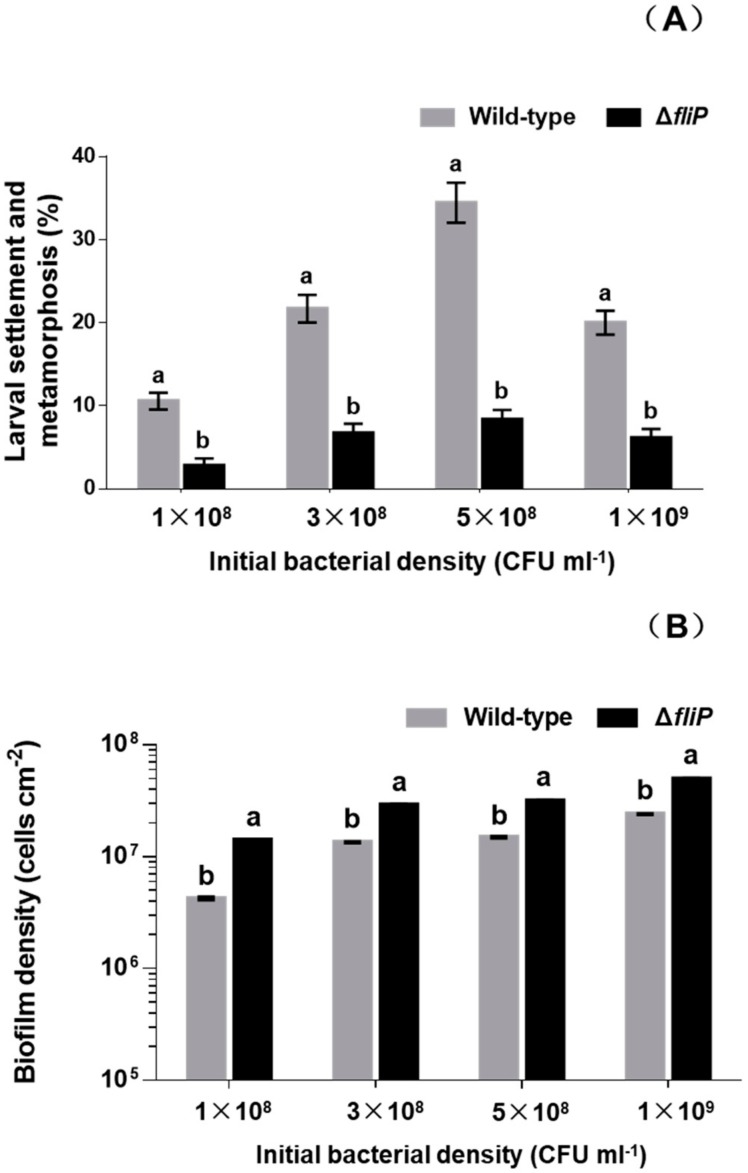
Relative to the wild-type strain the Δ*fliP* strain reduced the inducing activity and increased bacterial density. (**A**) Inducing activities of biofilms on larval settlement and metamorphosis with different initial colony-forming unit (CFU); (**B**) biofilm density on glass slips with different initial CFU. Tested biofilms formed on the glass slips for 48 h.

In order to test that the impact of deficiency of *fliP* gene on the mussel settlement-inducing activity, we constructed a flagellin synthetic protein gene *fliP* deletion mutant of *P. marina* (Figure 1) and compared the inducing activity of biofilms formed by wild-type and Δ*fliP* strains on the larvae of *M. coruscus* (Figure 2). The biofilms developed by the Δ*fliP* strain significantly reduced larval settlement and metamorphosis (*p* < 0.05, Figure 2A). At an initial concentration of 5 × 10^8^ colony-forming units (CFU)/mL, larval settlement and metamorphosis rate in biofilms formed by Δ*fliP* strain was only 8.33%, a 75.01% reduction compared with the wild-type strain (Figure 2A). In contrast, biofilm cell densities in the Δ*fliP* strain increased significantly compared to the wild-type strain in all tested concentrations (*p* < 0.05, Figure 2B).

### 2.2. Effects of fliP Gene Deletion on Flagellum Morphology and Structure to P. marina

**Figure 3:**
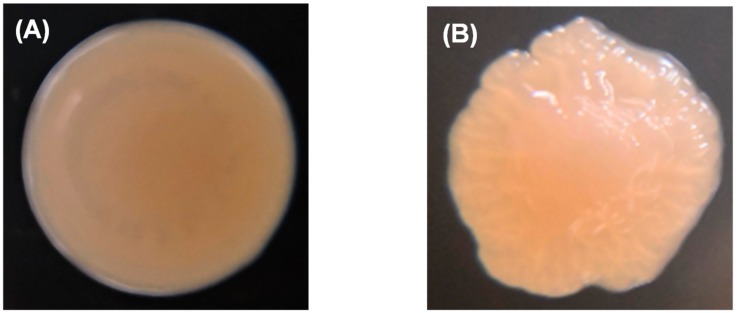

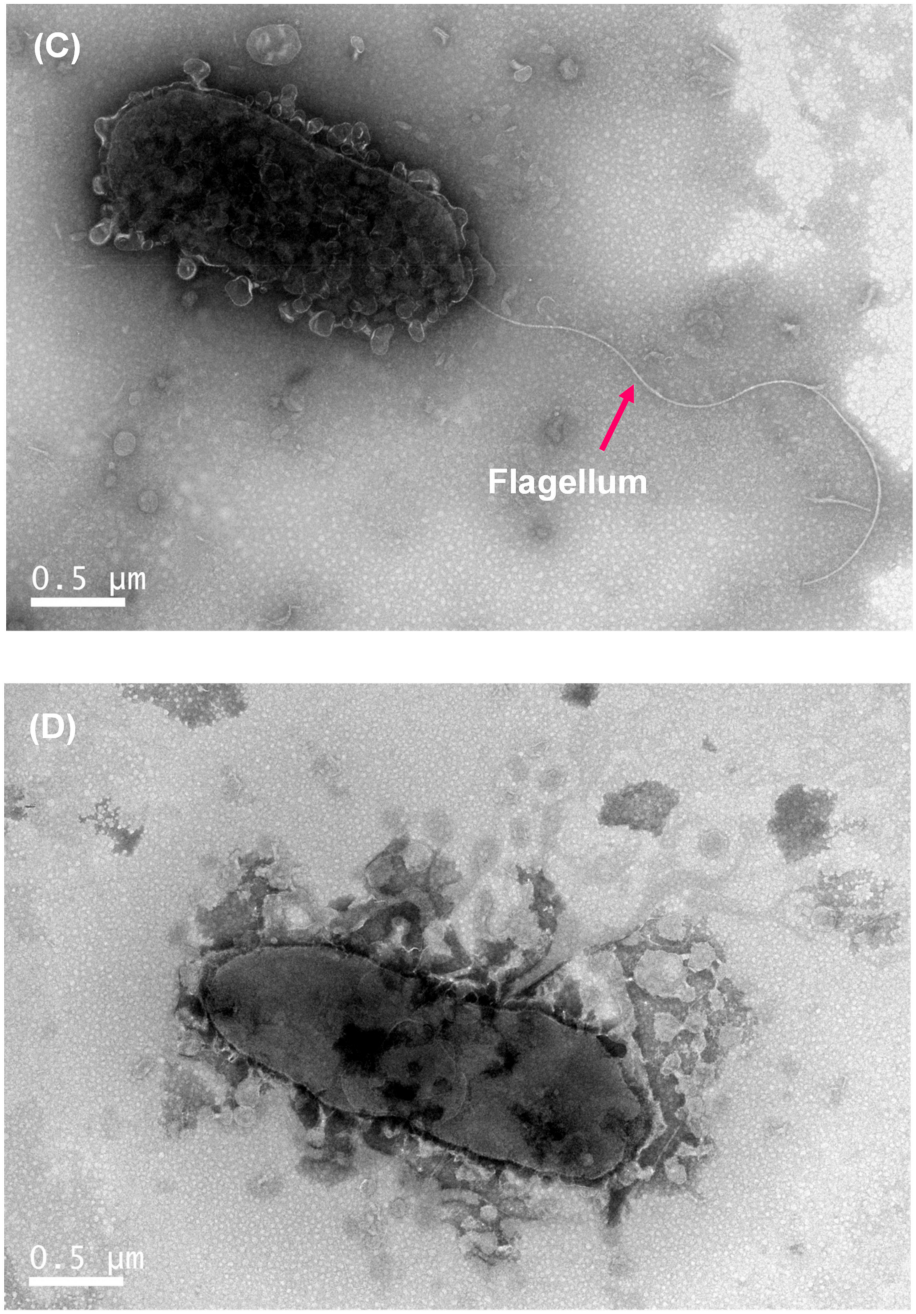
Colony morphology of wild-type (**A**) and Δ*fliP* (**B**) strains and transmission electron microscopy images of wild-type (**C**) and Δ*fliP* (**D**) strains.

The wild-type colony of *P. marina* showed a smooth and round shape (Figure 3A), while the Δ*fliP* mutant showed a wrinkled shape (Figure 3B). The results of transmission electron microscopy (TEM) revealed the monopolar flagellum of the wild-type strain (Figure 3C) and the absence of the flagellum in *fliP* deletion mutant (Figure 3D). 

### 2.3. Increased Biofilm Forming Ability after fliP Deletion

**Figure 4:**
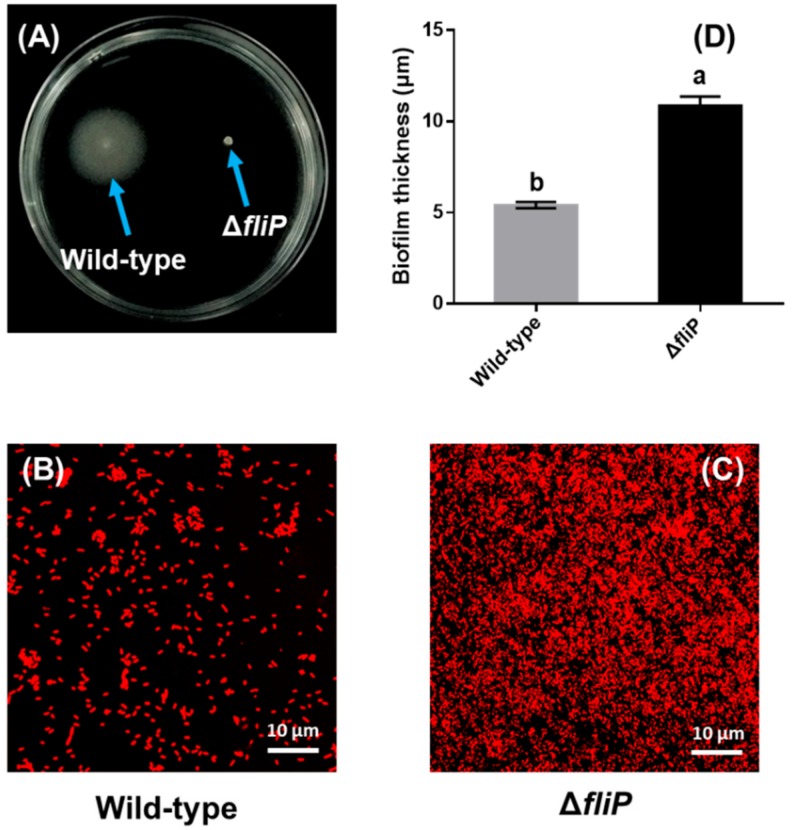
Biofilm formation of wild-type and Δ*fliP* strains. (**A**) Swimming motility of wild-type and Δ*fliP* strains; (**B**) the confocal laser scanning microscopy (CLSM) images of biofilms formed by the wild-type strain; (**C**) the CLSM images of Δ*fliP* biofilms; (**D**) biofilm thickness statistical analysis of wild-type and Δ*fliP* strains.

The *fliP* deletion mutant of *P. marina* lost motility compared to the wild-type strain (Figure 4A). A confocal laser scanning microscopy (CLSM) image showed that cell aggregation of biofilms formed by the Δ*fliP* strain was different from that of the wild-type strain (Figure 4B,C). The thickness of the biofilm formed by the Δ*fliP* strain increased more than onefold in comparison to the wild-type strain (*p* < 0.05, Figure 4D).

### 2.4. Variance of Biofilm EPS and Inducing Activities by Enzyme Treatment 

**Figure 5:**
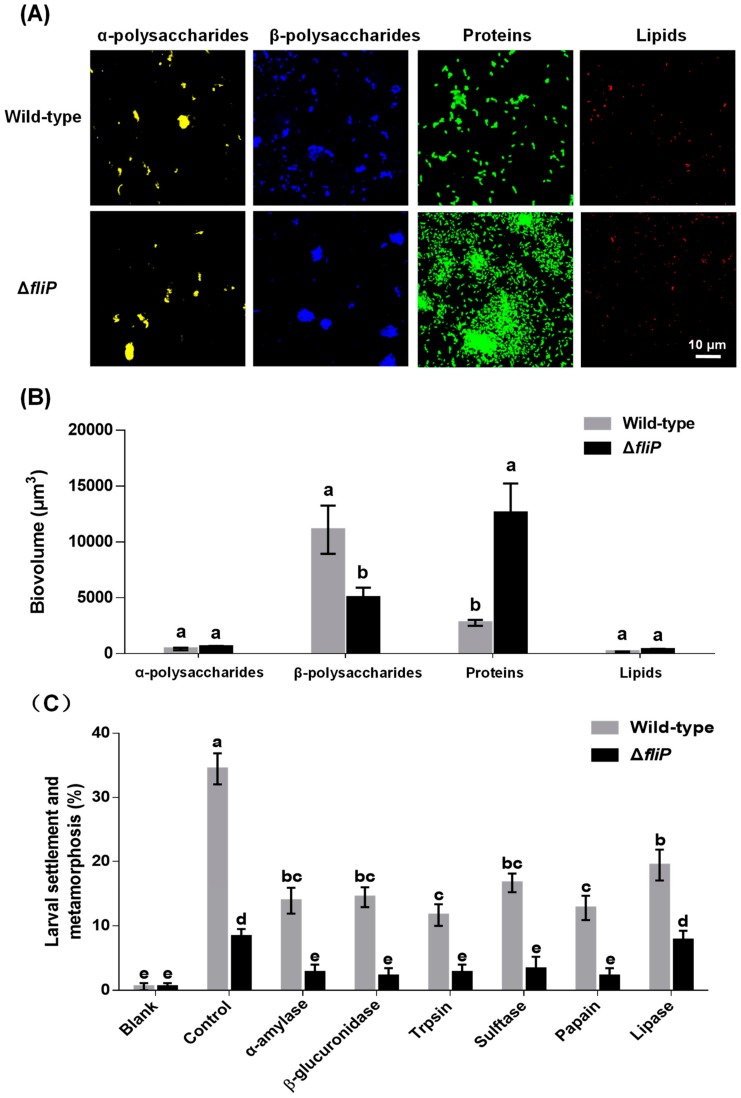
The CLSM analysis of *Pseudoalteromonas marina* biofilm EPS and mussel settlement and metamorphosis after enzyme treatments. (**A**) Distribution of polysaccharides, proteins and lipids in biofilms of wild-type and Δ*fliP* strains; (**B**) Biovolume analysis of extracellular polysaccharides, proteins and lipids content in biofilms; (**C**) Mussel settlement and metamorphosis on biofilms treated by different proteases, lipases and glycohydrolases.

CLSM images revealed that the distribution of β-polysaccharides and proteins on biofilms formed by the Δ*fliP* strain was different from that of the wild-type strain (Figure 5A). In the case of α-polysaccharides and lipids, no difference was observed between Δ*fliP* and wild-type strains (Figure 5A). The analysis of biovolume also showed a similar tendency to the result of CLSM (Figure 5B). The β-polysaccharide content in biofilms formed by the Δ*fliP* strain decreased 54.94% compared to that of the wild-type strain, and the protein content in biofilms formed by the Δ*fliP* strain increased more than threefold (*p* < 0.05, Figure 5B). Inducing activities of *P. marina* biofilms treated by trypsin, β-glucuronidase, α-amylase, sulfatase, papain, and lipase were reduced significantly (*p* < 0.05, Figure 5C). With the exception of lipase treatment, inducing activities of *P. marina* biofilms formed by the Δ*fliP* strain disappeared in other tested enzyme treatment groups compared to blank (*p* > 0.05, Figure 5C).

### 2.5. Effects of Flagellar Protein Extracted on Inducing Activities

**Figure 6:**
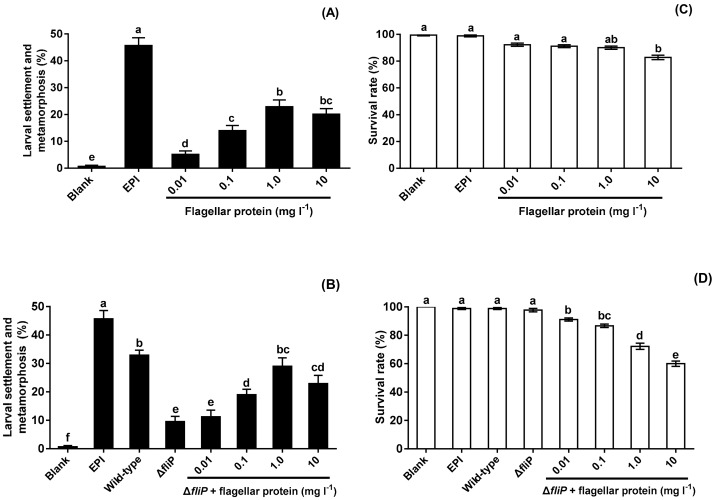
Effects of flagellar protein extracted on settlement and metamorphosis and survival rates. (**A**) Percentages of settlement and metamorphosis on flagellar protein tested; (**B**) percentages of settlement and metamorphosis on Δ*fliP* biofilms with addition of flagellar proteins; (**C**) effects of flagellar proteins extracted on larval survival rates; (**D**) effects of Δ*fliP* biofilms with addition of flagellar proteins extracted on larval survival rates.

No flagellar protein was extracted from the Δ*fliP* strain. Flagellar protein extracted from the wild-type strain promoted mussel pediveliger larvae to complete settlement and metamorphosis compared with blank (*p* < 0.05, Figure 6A). The inducing activities of flagellar protein increased and reached the maximum at the concentration of 1.0 mg L^−1^ (Figure 6A). Addition of flagellar protein resulted in significant increase of inducing activities of biofilms formed by the Δ*fliP* strain at 0.1, 1.0, and 10 mg L^−1^ (*p* < 0.05, Figure 6B). Furthermore, Δ*fliP* biofilms with added flagellar protein of 1.0 mg L^−1^ induced similar percentage of settlement and metamorphosis compared to biofilms formed by the wild-type strain (*p* > 0.05, Figure 6B). Larval survival rate only decreased significantly at 10 mg l^−1^ (*p* < 0.05, Figure 6C), In case of Δ*fliP* biofilms with addition of flagellar protein, larval survival rates decreased at all tested concentrations (*p* < 0.05, Figure 6D). 

## 3. Discussion

Marine biofilms play a key role in larval settlement and metamorphosis and establishment of macrofouling communities [3,4,5,6,24]. Here, we constructed a flagellin synthetic protein gene *fliP* deletion mutant of *P. marina* and demonstrated for the first time that the biofilms developed by the *fliP* deletion mutant could effectively reduce mussel settlement and metamorphosis.

Motility is a major dynamic feature in microbial worlds [25]. The bacterial flagellum could allow cells to swim and attach to the surfaces [26]. Thus, it is pivotal for the mobility of bacteria and subsequent biofilm formation [7,27,28,29]. Flagellar synthesis is accompanied by mediation of flagellar gene expression [30]. Here, we demonstrated that the *fliP* deletion causes the loss of the flagella structure and motility and subsequently facilitates more cell aggregation and biofilm formation. Previous work also revealed that inhibiting motility is helpful for stabilizing early cell aggregation during biofilm formation [31,32]. Although the mechanism of motility inhibition during biofilm formation remains poorly understood, one possible reason is due to conserving energy once the biofilm has formed [32]. In addition, other abiotic conditions of water need to be considered. 

A quantity of bacteria inhibits motility accompanied by the self-produced EPS matrix and formation of biofilms [31]. EPS that serve as nutrients also promote bacterial cells in close proximity [33]. Polysaccharides, proteins, nucleic acids, and lipids are major components of the EPS [33,34]. Here, we found that the Δ*fliP* strain produced fewer β-polysaccharides and more extracellular proteins during biofilm formation and exhibited no effect on producing α-polysaccharides and lipids. This suggests that β-polysaccharides and extracellular proteins contribute to the bacterial spreading and aggregation within biofilms and subsequently change the architecture of biofilms. 

Biofilms formed by bacteria have been known to regulate many invertebratesʼ settlement and metamorphosis [4]. *Pseudoalteromonas* is widespread in marine environments [12,23,35,36], and biofilms formed by *Pseudoalteromonas* spp. strains mediate invertebratesʼ settlement and metamorphosis [13,15,16,17,18,37,38,39,40]. Our previous works have demonstrated that biofilms formed by the genus *Pseudoalteromonas* trigger mussel settlement and metamorphosis [14,21,22,41]. The present findings revealed that deletion of flagellin synthetic protein gene *fliP* results in a decrease in the inducing activity of *P. marina* biofilms. Deletion of *fliP* gene leads to the loss of flagellum and motility of cells and increase in aggregation and density of cells. This suggests that cell density might not be a key factor regulating the musselʼs settlement and metamorphosis. Simultaneously, deletion of the *fliP* gene also resulted in a decrease of β-polysaccharides and increased extracellular proteins of biofilms. Another possible reason may be due to the lower production of the settlement-inducing substance by the mutant biofilm. In addition, treatment by the proteolytic enzymes and glycosidase also resulted in a decrease of inducing activity of biofilms formed by wild-type or Δ*fliP* strains. This further supports the idea that the polysaccharides and extracellular proteins in biofilms play crucial roles in mussel settlement and metamorphosis. In our previous work on *Mytilus galloprovincialis*, exopolysaccharide or glycoprotein on *Alteromonas* sp. 1 biofilms also was responsible for larval settlement and metamorphosis [42]. The finding is consistent with previous works on the polychaete, barnacle, and ascidian [43,44,45].

Bacterial flagellum is an important virulence factor that promotes tissue tropism in host–microbe interactions [46,47]. In addition, flagella also have a number of functions including adhesion or invasion of host cells, biofilm formation, and host development [47,48,49,50]. Flagellar protein, a principal component of bacterial flagella, could be recognized by the Toll-like receptor 5 (TLR5) of mice [49]. The present study suggested that flagellar protein was only extracted from wild-type *P. marina*, while no flagellar protein could be extracted from Δ*fliP* strain. Moreover, flagellar protein triggered larval metamorphosis of *M. coruscus,* and inducing activity depended on the concentrations. Simultaneously, adding flagellar protein into biofilms formed by the Δ*fliP* strain also increased and restored inducing activity on larval settlement and metamorphosis compared with that of the wild-type strain. In addition, we also found the existence of larval mortality, and this indicates that a sublethal toxic effect may result in the larval settlement and metamorphosis [51,52]. To date, only one piece of research on proteinaceous bacterial cues has been reported in stimulating larval settlement and metamorphosis in marine invertebrates [16]. Our findings demonstrated for the first time that flagellar proteins derived from *P. marina* promote mussel settlement and metamorphosis. Whether TLR5 in *M. coruscus* could recognize the flagellar protein of *P. marina* and finally cause the settlement and metamorphosis remains unknown. The mechanism governing the mussel–*Pseudoalteromonas* interaction needs to be uncovered.

## 4. Materials and Methods 

### 4.1. Strains, Plasmids, and Culture Conditions

*P. marina,* which induces both larval settlement and metamorphosis and plantigrade settlement of postlarvae in *M. coruscus,* was used [14,20]. Primers and plasmids are listed in Table S1. The *Escherichia coli* WM3064 and the plasmid pK18mobsacB-Ery used in this study were presented by Prof. Xiaoxue Wang, Institute of South China Sea of the Chinese Academy of Sciences. The *E. coli* WM3064 was grown at 37 °C in Luria-Bertani (LB) medium with 0.3 mM DAP (2,6-diamino-pimelic acid). The *P. marina* and Δ*fliP* strain were grown in Zobell 2216E agar plates at 25 °C. The *E. coli* WM3064 and *P. marina,* which harbored plasmid pK18mobsacB-Ery, were cultured in LB and Zobell 2216E medium with kanamycin (50 μg mL^−1^) or erythromycin (25 μg mL^−1^) as per the previously published method [21]. The Zobell 2216E agar plates were prepared by dissolving 5.0 g peptone, 1.0 g yeast extract, 0.01 g ferric citrate, and 15.0 g agar in 1 L seawater.

### 4.2. Construction of ΔfliP Strain

The Δ*fliP* mutant was constructed following the previously published method [21]. The upstream and downstream regions of the *fliP* were amplified by primers listed in Table S1. The restriction enzyme sites were added in the 5′ ends of polymerase chain reaction (PCR) products. Plasmids and PCR products were digested by different enzymes and constructed as recombination plasmids by ligation. The suicide plasmid was transferred to *E. coli* WM3064. The conjugal transfer between wild-type *P. marina* and *E.coli* WM3064 was performed at mating agar. The mating agar was prepared by 5.0 g peptone, 1.0 g yeast extract, 0.01 g ferric citrate, 15.0 g agar, 0.3 mM DAP, 500 mL sea water, and 500 mL distilled water. After 2–5 days’ mating, the recombinant plasmids were integrated into the *P. marina* chromosome. Bacteria that could grow on Zobell 2216E medium with 25 μg mL^−1^ erythromycin were picked. The single-crossover strains were verified by primer *fliP*–SF/*fliP*–LR or *fliP*–LF/*fliP*–SR. and platted on 2216E medium with 15% sucrose, and Δ*fliP* were screened by primer sets *fliP*–SF/*fliP*–LR, *fliP*–LF/*fliP*–SR, *fliP*–SF/*fliP*–SR, *fliP*–LF/*fliP*–LR.

### 4.3. The Acquisition of Larvae 

Two-year-old *M. coruscus* were collected from Gouqi island, China (122°44′ E; 30°73′ N). Spawning was performed following previously published methods [14,19,53]. After 2 days, the swimming straight-hinge veliger larvae were acquired. These larvae were cultured at 18 °C in glass beakers (5 larvae mL^−1^) without light. The cultural water was replaced by fresh filtered seawater (FSW; pore size: 0.45 μm) every two days, and the diet of larvae was *Isochrysis zhanjiangensis*. The larvae developed to pediveliger stage were used for settlement and metamorphosis bioassays.

### 4.4. The Bioassay of Larval Settlement and Metamorphosis 

Biofilms of wild-type and Δ*fliP* strains were formed following a method modified from Yang et al. [14]. Briefly, *P. marina* and Δ*fliP* strain were inoculated in Zobell 2216E medium and cultured at 25 °C for 16–18 h with shaking 200 rpm. The cells were centrifuged at 1600× *g* for 15 min. The supernatant was removed, and the cells of precipitate were washed three times by autoclaved filtered (pore size: 0.45 μm) seawater (AFSW). The initial bacterial density was diluted to 1 × 10^8^, 3 × 10^8^, 5× 10^8^, and 1× 10^9^ colony-forming unit (CFU) mL^−1^ and added into each Petri dish (Ø 64 mm × 19 mm height). A glass slip (size: 38 mm × 26 mm) was contained in each Petri dish. Biofilms formed on the glass slip at 18 °C for 48 h. Unattached bacteria were removed by washing three times with AFSW. Twelve washed biofilms were used for the settlement and metamorphosis bioassays (see below). 

To investigate the bioactive moiety of the inductive cues of biofilms formed by *P. marina* and Δ*fliP* strain, different enzyme treatments were performed following a previously published method [54,55]. Tris-HCl (10 mM, pH = 7) was added into 50% glycerol for preparing the enzymes’ stock solutions. Working solutions were prepared by diluting with AFSW. The information on enzymes is listed in Table S2. Biofilms were incubated in an enzyme solution at 25 °C for 3 h. Treated biofilms were washed with AFSW three times. Nine treated biofilms were used for larval settlement and metamorphosis bioassays (see below).

Each glass Petri dish, which contained 20 mL AFSW, a glass slip with biofilm, and 20 pediveliger larvae, was used for bioassay. Petri dishes were cultured at 18 °C without light. After 48 h, the percentages of settlement and metamorphosis were used for evaluating the inducing activity of biofilms. The Olympus stereoscopic microscope was used to observe larval behavior and body structure change. A clean glass slip without biofilm was served as a negative control.

### 4.5. Swimming Motility Determination

The wild-type and Δ*fliP* strains were cultured in Zobell 2216E liquid medium at 25 °C for 12 h. One microliter of bacterial supernatant was dropped on Zobell 2216E medium (0.3% agar). This experiment was performed at 25 °C. After 16–18 h incubation, the migration zone diameter as an index to evaluate the motility was measured. This experiment was repeated three times.

### 4.6. Transmission Electron Microscopy (TEM) 

The wild-type and ΔfliP strains were cultured in Zobell 2216E liquid medium at 25 °C for 16–18 h. Bacterial strains were observed by TEM following the published method [21]. The bacterial culture was dropped on a formvar-coated mesh membrane. The bacterial suspension was maintained at membrane for 2 min. The membrane was stained by phosphotungstic acid (30 g L−1, pH = 7.0) for 2 min. The stained membrane was air-dried and observed by TEM (Tecnai G2 SpiritBiotwin, FEI, Oregon, USA).

### 4.7. Detection of Bacterial Density of Biofilms

The 5% formalin solution was used to fix biofilms. After 48 h of fixing, biofilms were stained by the acridine orange solution (0.1%, g/v) for 5 min. Bacterial density (cells cm^−2^) was calculated by an Olympus BX51 microscope (Tokyo, Japan) at 1000× magnification. Ten random fields of views were selected from each biofilm. The assay had three independent replications. 

### 4.8. The Inductive Effect of Extracted Flagellar Proteins 

In this study, the flagellar protein was extracted by a modified method of acid hydrolysis ultracentrifugation [56]. The wild-type strains of *P. marina* were incubated in 1000 mL Zobell 2216E. In the case of the Δ*fliP* strain, mutants were incubated in 1000 mL, 2000 mL, and 5000 mL of Zobell 2216E, respectively. Bacteria were cultured at 25 °C with shaking of 80 r/min for 18 h. Bacterial cultures were centrifuged at 4000 rpm for 15 min. The bacterial cells were collected from precipitates and washed with AFSW three times. Washed bacterial cells were resuspended in autoclaved physiological saline (0.9% sodium chloride dissolved in distilled water) and adjusted to pH 2.0 using HCl (1 mol L^−1^). After 30 min of magnetic stirring (600 rpm), bacterial suspension was centrifuged at 10,000× *g*, 4 °C for 1 h. The supernatant containing flagellar protein was collected. The pH value of supernatant was adjusted to 7.2 by adding NaOH (1 mol L^−1^). The supernatant was then mixed with ammonium sulphate (Sangon, Shanghai, China) to a final concentration of 2.67 mol L^−1^. The mixture was placed at 4 °C for 24 h. Flagellar proteins were collected from precipitates by centrifugal at 15,000× *g*, 4 °C, 15 min. Flagellar proteins were resuspended in 5 mL autoclaved distilled water and dialyzed against autoclaved distilled water for 2 h with magnetic stirring (600 rpm). The dialyzed bags were placed in glass breakers which contained 2 L autoclaved distilled water and 20 g activated carbon. The protein concentration was measured using BradFord assay by kit (Sangon, Shanghai, China). The extracted flagellar protein from wild-type strains were stored at −20 °C. 

The final concentrations of the extracted flagellar protein suspension were adjusted to 0.01, 0.1, 1.0, and 10 mg L^−1^. Each Petri dish contained 20 mL of the extracted flagellar proteins suspension and 20 pediveligers. The biofilms of the Δ*fliP* mutant were added in extracted flagellar proteins suspension to examine the inductive effect. The biofilm of wild-type and Δ*fliP* strains were set as positive controls. The AFSW was set as negative controls. 

### 4.9. Analysis of Confocal Laser Scanning Microscopy (CLSM) Images of Biofilms

Biofilms were stained following the method of González-Machado et al. [57]. Details of used fluorescent dyes are listed in Table S3. Staining solutions were diluted by 150 mM NaCl. The staining solution was added on biofilms of *P. marina* and Δ*fliP* strain for 20 min without light. The stained biofilms were washed with NaCl at 150 mM and photographed by CLSM (Leica TCS SP8, Japan). The CLSM images were acquired by the LAS X Version (Leica, Weztlar, Germany) at pixels of 1024 × 1024 and z-step of 0.20 μm. The Image J software was used to calculate for threshold value (National Institutes of Health, Bethesda, Maryland, USA). The threshold value was converted to biovolumes (μm^3^). Three fields of vision were selected from each biofilm. Three biological repetitions of staining were set for each biofilm. 

### 4.10. Data Statistical analysis

The percentage of settlement and metamorphosis was arcsine transformed and tested the normality of data by Shapiro–Wilk’s W test. The Kruskal–Wallis followed by the Steel-Dwass All Pairs test was used to determine significant differences. The correlations analysis was performed by Spearman’s rank correlation test. Data processing was performed by JMP^TM^ software (version 10.0.0, SAS, North Carolina, USA).

## 5. Conclusions

Taken together, the present study shows that the deletion of flagellin synthetic protein gene *fliP* causes the loss of the flagella structure and motility of the Δ*fliP* mutant of *P. marina*. These changes in the aflagellate strain promote the aggregation of biofilm bacterial cells by reducing β-polysaccharides and increasing extracellular proteins and reduce mussel settlement and metamorphosis. Flagellar protein extracted from wild-type strain of *P. marina* could promote mussel metamorphosis, and Δ*fliP* biofilms combined with additional flagellar protein restored inducing activity as the same level as that of the wild-type strain. These findings contribute to our understanding of the molecular interactions between the biofilm and mussel settlement and metamorphosis. 

## Supplementary Materials

Supplementary materials can be found at https://www.mdpi.com/1422-0067/21/3/710/s1.

## References

[1] Dang H., Lovell C.R. (2016). Microbial surface colonization and biofilm development in marine environments. Microbiol. Mol. Biol. Rev..

[2] Flemming H.C., Wingender J., Szewzyk U., Steinberg P., Rice S.A., Kjelleberg S. (2016). Biofilms: An emergent form of bacterial life. Nat. Rev. Microbiol..

[3] Dobretsov S., Flemming H.C., Murthy P.S., Venkatesan R., Cooksey K. (2009). Inhibition and induction of marine biofouling by biofilms. Marine And Industrial Biofouling.

[4] Hadfield M.G. (2011). Biofilms and marine invertebrate larvae: What bacteria produce that larvae use to choose settlement sites. Annu. Rev. Mar. Sci..

[5] Salta M., Capretto L., Carugo D., Wharton J.A., Stokes K.R. (2013). Life under flow: A novel microfluidic device for the assessment of anti-biofilm technologies. Biomicrofluidics.

[6] Qian P.Y., Dahms H.U., Flemming H.C., Murthy P.S., Venkatesan R., Cooksey K. (2009). A triangle model: Environmental changes affect biofilms that affect larval settlement. Marine and Industrial Biofouling.

[7] Lema K.A., Constancias F., Rice S.A., Hadfield M.G. (2019). High bacterial diversity in nearshore and oceanic biofilms and their influence on larval settlement by Hydroides elegans (Polychaeta). Environ. Microbiol..

[8] Fulaz S., Vitale S., Quinn L., Casey E. (2019). Nanoparticle–biofilm interactions: The role of the EPS matrix. Trends. Microbiol..

[9] Railkin A., Tatiana G., Oleg M. (2003). Marine Biofouling: Colonization Processes and Defenses.

[10] Zhang W., Ding W., Li Y.X., Tam C., Bougouffa S., Wang R., Pei B., Chiang H., Leung P., Lu Y. (2019). Marine biofilms constitute a bank of hidden microbial diversity and functional potential. Nat. Commun..

[11] Flemming H.C., Wuertz S. (2019). Bacteria and archaea on earth and their abundance in biofilms. Nat. Rev. Microbiol..

[12] Gauthier G., Gauthier M., Christen R. (1995). Phylogenetic analysis of the genera *Alteromonas*, *Shewanella* and *Moritella* using genes-coding for small-subunit ribosomal-RNA sequences and division of the genus *Alteromonas* into 2 genera, *Alteromonas* (Emended) and *Pseudoalteromonas* gen-nov, and proposal of 12 new species combinations. Inter. J. Syst. Bacteriol..

[13] Holmström C., Egan S., Franks A., McCloy S., Kjelleberg S. (2002). Antifouling activities expressed by marine surface associated *Pseudoalteromonas* species. FEMS Microbiol. Ecol..

[14] Yang J.L., Shen P.J., Liang X., Li Y.F., Bao W.Y., Li J.L. (2013). Larval settlement and metamorphosis of the mussel *Mytilus coruscus* in response to monospecific bacterial biofilms. Biofouling.

[15] Shikuma N.J., Pilhofer M., Weiss G.L., Hadfield M.G., Jensen G.J., Newman D.K. (2014). Marine tubeworm metamorphosis induced by arrays of bacterial phage tail–like structures. Science.

[16] Ericson C.F., Eisenstein F., Medeiros J.M., Malter K.E., Cavalcanti G.S., Zeller R.W., Newman D.K., Pilhofer M., Shikuma N.J. (2019). A contractile injection system stimulates tubeworm metamorphosis by translocating a proteinaceous effector. Elife.

[17] Huang Y., Callahan S., Hadfield M.G. (2012). Recruitment in the sea: Bacterial genes required for inducing larval settlement in a polychaete worm. Sci. Rep..

[18] Shikuma N.J., Antoshechkin I., Medeiros J.M., Pilhofer M., Neman D.K. (2016). Stepwise metamorphosis of the tubeworm *Hydroides elegans* is mediated by a bacterial inducer and MAPK signaling. Proc. Natl. Acad. Sci. USA.

[19] Wang C., Bao W.Y., Gu Z.Q., Li Y.F., Liang X., Ling Y., Cai S.L., Shen H.D., Yang J.L. (2012). Larval settlement and metamorphosis of the mussel *Mytilus coruscus* in response to natural biofilms. Biofouling.

[20] Li Y.F., Guo X.P., Yang J.L., Liang X., Bao W.Y., Shen P.J., Shi Z.Y., Li J.L. (2014). Effects of bacterial biofilms on settlement of plantigrades of the mussel *Mytilus coruscus*. Aquaculture.

[21] Zeng Z.S., Guo X.P., Li B.Y., Wang P.X., Cai X.S., Tian X.P., Zhang S., Yang J.L., Wang X.X. (2015). Characterization of self-generated variants in *Pseudoalteromonas lipolytica* biofilm with increased antifouling activities. Appl. Microbiol. Biotechnol..

[22] Zeng Z., Guo X.P., Cai X., Wang P., Li B., Yang J.L., Wang X. (2017). Pyomelanin from *Pseudoalteromonas lipolytica* reduces biofouling. Microb. Biotechnol..

[23] Peng L.H., Liang X., Guo X.P., Yoshida A., Osatomi K., Yang J.L. (2018). Complete genome of *Pseudoalteromonas marina* ECSMB14103, a mussel settlement-inducing bacterium isolated from the East China Sea. Mar. Genom..

[24] Wieczorek S.K., Todd C.D. (1998). Inhibition and facilitation of settlement of epifaunal marine invertebrate larvae by microbial biofilm cues. Biofouling.

[25] Son K., Brumley D.R., Stocker R. (2015). Live from under the lens: Exploring microbial motility with dynamic imaging and microfluidics. Nat. Rev. Microbiol..

[26] Macnab R.M. (2003). How bacteria assemble flagella. Annu. Rev. Microbiol..

[27] O’Toole G.A., Kolter R. (1998). Flagellar and twitching motility are necessary for *Pseudomonas aeruginosa* biofilm development. Mol. Microbiol..

[28] Watnick P.I., Lauriano C.M., Klose K.E., Croal L., Kolter R. (2001). The absence of a flagellum leads to altered colony morphology, biofilm development and virulence in *Vibrio cholerae* O139. Mol. Microbiol..

[29] Lemon K.P., Higgins D.E., Kolter R. (2007). Flagellar motility is critical for *Listeria monocytogenes* biofilm formation. J. Bacteriol..

[30] Barker C.S., Meshcheryakova I.V., Inoue T., Samatey F.A. (2014). Assembling flagella in *Salmonella* mutant strains producing a type III export apparatus without FliO. J. Bacteriol..

[31] Guttenplan S.B., Blair K.M., Kearns D.B. (2010). The EpsE flagellar clutch is bifunctional and synergizes with EPS biosynthesis to promote *Bacillus subtilis* biofilm formation. PLoS Genet..

[32] Subramanian S.B., Kearns D.B. (2019). Functional regulators of bacterial flagella. Annu. Rev. Microbiol..

[33] Flemming H.C., Wingender J. (2010). The biofilm matrix. Nat. Rev. Microbiol..

[34] Koo H., Allan R.N., Howlin R.P., Stoodley P., Hall-Stoodley L. (2017). Targeting microbial biofilms: current and prospective therapeutic strategies. Nat. Rev. Microbiol..

[35] Qin Q.L., Li Y., Zhang Y.J., Zhou Z.M., Zhang W.X., Chen X.L., Zhang X.Y., Zhou B.C., Wang L., Zhang Y.Z. (2011). Comparative genomics reveals a deep-sea sediment-adapted life style of *Pseudoalteromonas* sp. SM9913. ISME J..

[36] Wang J.S., Peng L.H., Guo X.P., Yoshida A., Osatomi K., Li Y.F., Yang J.L., Liang X. (2019). Complete genome of *Pseudoalteromonas atlantica* ECSMB14104, a Gammaproteobacterium inducing mussel settlement. Mar. Genom..

[37] Dahms H., Dobretsov S., Qian P. (2004). The effect of bacterial and diatom biofilms on the settlement of the bryozoan Bugula Neritina. J. Exp. Mar. Biol. Ecol..

[38] Huggett M.J., Williamson J.E., de Nys R., Kjelleberg S., Steinberg P.D. (2006). Larval settlement of the common Australian sea urchin *Heliocidaris erythrogramma* in response to bacteria from the surface of coralline algae. Oecologia.

[39] Tebben J., Tapiolas D.M., Motti C.A., Abrego D., Negri A.P., Blackall L.L., Steinberg P.D., Harder T. (2011). Induction of larval metamorphosis of the coral *Acropora millepora* by tetrabromopyrrole isolated from a *Pseudoalteromonas* bacterium. PLoS ONE.

[40] Sneed J.M., Sharp K.H., Ritchie K.B., Paul V.J. (2014). The chemical cue tetrabromopyrrole from a biofilm bacterium induces settlement of multiple Caribbean corals. Proc. Biol. Sci..

[41] Sun J.J., Liang X., Guo X.P., Chen Y.R., Ding D.W., Zhang D.M., Yang J.L. (2016). Effects of culture media on the biofilm formation and subsequent settlement of *Mytilus coruscus*. J. Fish. China.

[42] Bao W.Y., Yang J.L., Satuito C.G., Kitamura H. (2007). Larval metamorphosis of the mussel *Mytilus galloprovincialis* in response to *Alteromonas* sp. 1: Evidence for two chemical cues?. Mar. Biol..

[43] Kirchman D., Graham S., Reish D., Mitchell R. (1982). Lectins may mediate in the settlement and metamorphosis of *Janua* (*Dexiospira*) *brasiliensis* Grube (Polychaeta: Spirorbidae). Mar. Biol. Lett..

[44] Maki J.S., Mitchell R. (1985). Involvement of lectins in the settlement and metamorphosis of marine invertebrate larvae. Bull. Mar. Sci..

[45] Khandeparker L., Anil A.C., Raghukumar S. (2003). Barnacle larval destination: Piloting possibilities by bacteria and lectin interaction. J. Exp. Mar. Biol. Ecol..

[46] Brennan C.A., Hunt J.R., Kremer N., Krasity B.C., Apicella M.A., McFall-Ngai M.J., Ruby E.G. (2014). A model symbiosis reveals a role for sheathed-flagellum rotation in the release of immunogenic lipopolysaccharide. Elife.

[47] Aschtgen M.S., Brennan C.A., Nikolakakis K., Cohen S., McFall-Ngai M., Ruby E.G. (2019). Insights into flagellar function and mechanism fromthe squid–vibrio symbiosis. NPJ Biofilms. Microbi..

[48] Young G.M., Badger J.L., Miller V.L. (2000). Motility is required to initiate host cell invasion by *Yersinia enterocolitica*. Infect. Immun..

[49] Hayashi F., Smith K.D., Ozinsky A., Hawn T.R., Yi E.C., Goodlett D.R., Eng J.K., Akira S., Underhill D.M., Aderem A. (2001). The innate immune response to bacterial flagellin is mediated by Toll-like receptor 5. Nature.

[50] Aschtgen M.-S., Wetzel K., Goldman W., McFall-Ngai M., Ruby E. (2016). Vibrio fischeri-derived outer membrane vesicles trigger host development. Cell. Microbiol..

[51] Dubilier N. (1988). H_2_S-a settlement cue or a toxic substance for Capitella sp. I larvae?. Biol. Bull..

[52] Yang J.L., Satuito C.G., Bao W.Y., Kitamura H. (2008). Induction of metamorphosis of pediveliger larvae of the mussel Mytilus galloprovincialis Lamarck, 1819 using neuroactive compounds, KCl, NH_4_Cl and organic solvents. Biofouling.

[53] Liang X., Chen K., Li Y.F., Bao W.Y., Yoshida A., Osatomi K., Yang J.L. (2019). An α_2_-adrenergic receptor is involved in larval metamorphosis in the mussel, *Mytilus coruscus*. Biofouling.

[54] Harder T., Dobretsov S., Qian P.Y. (2004). Waterborne polar macromolecules act as algal antifoulants in the seaweed *Ulva reticulata*. Mar. Ecol. Prog. Ser..

[55] Jaffar N., Ishikawa Y., Mizuno K., Okinaga T., Maeda T. (2016). Mature biofilm degradation by potential probiotics: *Aggregatibacter actinomycetemcomitans* versus *lactobacillus* spp.. PLoS ONE.

[56] Ibrahim G.F., Fleet G.H., Lyons M.J., Walker R.A. (1985). Method for the isolation of highly purified *Salmonella* flagellins. J. Clin. Microbiol..

[57] González-Machado C., Capita1 R., Riesco-Peláez F., Alonso-Calleja C. (2018). Visualization and quantification of the cellular and extracellular components of Salmonella Agona biofilms at different stages of development. PLoS ONE.

